# Obesity and the Heart: Webcast March 18 2025

**DOI:** 10.14797/mdcvj.1604

**Published:** 2025-05-01

**Authors:** Miguel A. Quiñones, Eleanora Avenatti, Zulqarnain Javed, Adi Lador, Ramiro Saavedra, Bhargavi Patham, Andres Calderon, Krishnaswami Vijayaraghavan, Nishtha Sareen

**Affiliations:** 1 Houston Methodist DeBakey Heart & Vascular Center, Houston, Texas, US; 2 Division of Cardiac Electrophysiology, Houston Methodist Hospital, US; 3 Center for Weight Loss and Bariatric Surgery, Houston Methodist Hospital, Houston, Texas, US; 4 Houston Methodist Hospital, Houston, Texas, US; 5 University of Arizona, Phoenix, Arizona, US; 6 Ascension St. Thomas Hospital, Nashville, Tennessee, US; 7 College of Medicine, University of Tennessee Health Science Center, Nashville, Tennessee, US

**Keywords:** obesity, weight loss, social determinants of health, cardiometabolic syndrome, fat mass disease, adiposopathy, diabesity

## Abstract

This 76-minute webcast features a conversation about “Obesity and the Heart”—the focus of Issue 21.2. Led by the issue’s editor, the discussion engages the authors on emerging themes and lessons learned while researching and writing the articles. View the video at https://vimeo.com/event/4867850.

**Video 1:** The editors and authors discuss details and explore overarching themes in Issue 21.2, titled “Obesity and The Heart.” View it at https://vimeo.com/event/4867850.

## Introduction

This 76-minute webcast (Video 1) features the *Journal*’s editor-in-chief, Dr. Miguel Quiñones, and the issue’s guest editor, Dr. Eleanora Avenatti, conversing with seven of the authors on the emerging themes and lessons learned while exploring the article topics related to “Obesity and the Heart.” The following list outlines where to view guests and topics:

### Welcome

Miguel Quiñones, MD, introduces topic (2:40)

Dr. Quiñones introduces guest editor Eleanora Avenatti, MD (4:31)

Dr. Avenatti explains overview of topics (5:10)

### Dr. Avenatti Begins Discussions

**Obesity and Atherosclerotic Cardiovascular Disease: A Review of Social and Biobehavioral Pathways (6:12)**

Dr. Avenatti introduces Zulqarnain Javed, PhD, MBBS, MPH (6:26)

Dr. Javed begins (7:00)

Dr. Quiñones comments (18:33)

Dr. Avenatti comments (19:31)

**Obesity and Atrial Fibrillation: A Comprehensive Review (20:40)**

Dr. Avenatti introduces Adi Lador, MD (20:41)

Dr. Lador begins (20:53)

Dr. Avenatti comments (29:12)

**Strategies to Manage Obesity: Lifestyle (32:00)**

Dr. Avenatti introduces Ramiro Saavedra, MS, RD, CSR, LD (32:25)

Ramiro Saavedra begins (33:00)

Dr. Avenatti comments (40:42)

**Medical Management of Obesity: Current Trends and Future Perspectives (42:20)**

Dr. Avenatti introduces Bhargavi Patham, MD, PhD (42:16)

Dr. Patham introduces Andres Calderon, MD (43:02)

Dr. Calderon begins (43:49)

Dr. Avenatti comments (53:08)

Dr. Patham begins (53:50)

**Food Chain and Food Policies: Causes and Solutions for the Obesity Pandemic (56:45)**

Dr. Avenatti introduces Krishnaswami Vijayaraghavan, MD, MS, and Nishtha Sareen, MD, MPH (57:10)

Dr. Vijayaraghavan begins (58:00)

Dr. Sareen focuses on Gender Disparity (1:05:25)

Dr. Vijayaraghavan comments (1:09:00)

## Conclusion

Dr. Avenatti wrap-up (1:14:52)

Dr. Quiñones wrap-up (1:15:35)

